# The Impact of Test Conditions and Lay-Up Configuration on SEA in Carbon–Epoxy Tubes

**DOI:** 10.3390/ma19194226

**Published:** 2026-10-05

**Authors:** Grażyna Ryzińska, Roman Gieleta, Jacek Tarasiuk, Sebastian Wroński

**Affiliations:** 1 Department of Materials Forming and Processing, The Faculty of Mechanical Engineering and Aeronautics, Rzeszow University of Technology, 35-029 Rzeszow, Poland; 2 Department of Materials Strength and Experimental Research, Institute of Mechanics and Computational Engineering, Faculty of Mechanical Engineering, Military University of Technology, 00-909 Warsaw, Poland; roman.gieleta@wat.edu.pl; 3 Department of Condensed Matter Physics, Faculty of Physics and Applied Computer Science, AGH University, 30-055 Krakow, Polandwronski.sebastian@gmail.com (S.W.)

**Keywords:** SEA, progressive crushing, crashworthiness, CFRP, SHPB

## Abstract

This article presents the effect of test velocity and the percentage of unidirectional (UD) and woven (WF) fibers on specific energy absorption (SEA). Composite tubes with an internal diameter of 20 mm were fabricated using five prepreg layers. Progressive crushing of the specimens was performed at speeds of 20 mm/min, 4.48–6.7 m/s, and 21 m/s. Crushing at a speed of 21 m/s has been shown to result in a 59–61% decrease in SEA relative to quasi-static conditions, depending on the specimen architecture. Computed tomography scans are included to show the shape and nature of the fragments after crushing and allow us to conclude that increasing the process velocity increases the average layer thickness after the crushing process, resulting in a reduction in the absorbed energy.

## 1. Introduction

Today, automotive and aircraft structures are subjected to increasingly higher loads, while safety requirements continue to rise. It is also desirable for modern structures to become increasingly lighter for reasons of fuel consumption and environmental protection. Composite materials are becoming increasingly popular as standalone structures and for the production of impact-energy-absorbing components [1]. Among the entire range of composite materials, fiber-reinforced composites [2,3,4,5,6,7,8,9,10] are the best energy-absorbing materials, according to recent research, while also offering a good stiffness-to-weight ratio [5,7,11,12,13,14,15].

The process of designing impact-energy-absorbing components requires key information on the factors influencing energy absorption. One of the most important factors is velocity and its effect on the amount of energy absorbed [16]. A review of the literature shows that experimental studies have provided results indicating both an increase in the amount of energy absorbed with increasing velocity, e.g., [17,18,19] (Table 1), and a decrease in the amount of energy absorbed with increasing velocity, e.g., [20,21,22,23,24]. In some cases, the result of absorbed energy was correlated with the geometry of the composite element. In the case of the bar test, an increase in energy was obtained, while in the case of the tube test, no change in the amount of energy absorbed was observed [18].

Analysis of the research results presented in [25,26] shows that the change in the amount of absorbed energy can be explained by differences in the coefficient of friction, and in the velocity range of 0.0002 m/s–8 m/s, both a decrease in energy (−15%) and an increase of up to 20% were recorded. A similar reason for changes in the amount of absorbed energy was recorded in [14,27]. The range of tested velocity was 0.001 m/s–12 m/s. These studies indicated that the coefficient of friction decreases with increasing velocity, which causes a decrease in the amount of absorbed energy. A change in the crushing mechanism was also indicated. The change in the crushing mechanisms is also a significant factor influencing energy absorption, as indicated in [22]. For high velocity, Local Fragmentation Failure (LFF) was identified, as opposed to Global Spanning Failure (GSF), which was dominant for low test velocity.

Sensitivity to strain rate and composite architecture was also demonstrated in studies [28,29,30,31,32,33], which showed a reduction in the number of microcracks at high strain rates.

In the work [32] the composite strength increased with increasing strain rate, while it decreased with increasing temperature. Failure mechanisms such as fiber breakage and matrix cracking were identified, enabling the characterization of the dynamic response of the plain-weave carbon–epoxy composite. The results of study [33] demonstrated that the tensile strength of CFRP laminates depends on the strain rate, whereas Young’s modulus remained insensitive to it. Furthermore, it was found that the effect of strain rate is linked to the stacking sequence and fiber orientation. Experimental results indicated that the strain rate effect is less pronounced in cross-ply laminates than in unidirectional laminates. Failure morphology analysis revealed that the differences in strain rate response among cross-ply, quasi-isotropic, and unidirectional laminates stemmed from a lack of compatibility between fiber and matrix behavior as the strain rate increased. The results of study [34] demonstrated that the material failure mechanisms along the principal direction depended on the strain rate. It was observed that as the strain rate increased—from quasi-static to high values—the failure mechanism along the fiber orientation shifted from crushing (accompanied by the formation of fiber kink bands) to mechanisms dominated by interlaminar delamination, longitudinal splitting, and delamination caused by fiber buckling. The results of study [35] showed no dependence of tensile strength, elastic modulus, or strain at failure on the strain rate.

Research conducted by various scientists indicates the need to systematize the testing methodology for composite materials for impact energy absorption. Among the methods used so far, such as compression of various types of open profiles, flat plates, and pipes with different cross-sectional shapes, the results obtained for tubular specimens are the most useful. The highest energy amounts are obtained using tubular elements, assuming that the wall thickness to diameter ratio (t/D) is in the range of 0.025–0.066 [36]. Therefore, it is advisable to conduct tests on composites intended for impact energy absorption using pipes with a specific t/D. To create test conditions that are closest to reality, it is beneficial to use an additional element, such as an initiator with appropriate geometry, and to properly prepare the specimen edge by shaping it, which triggers the crushing process, e.g., in the form of a chamfer with an appropriate inclination angle.

Works [37,38,39] point to another factor that is crucial in the energy absorption process of composite structures: the type of reinforcement. Work [40] demonstrated that long fiber reinforcement yields better results compared to short fibers and mats. Furthermore, work [24] examined the effect of reinforcement architecture on the amount of energy absorbed for a specific geometry of tubular specimens. To confirm these conclusions, tests should be conducted for other specimen geometries, materials, and velocities. This would enable the use of the obtained data in the design of such components.

The behavior of the matrix plays a crucial role in the analysis of the composite’s overall behavior as a function of test speed.

As a widely used matrix in fiber-reinforced polymer composites, epoxy resins play a key role in determining the impact resistance of these materials. Study [41] investigated the effect of ambient temperature on the mechanical properties of epoxy resin at various strain rates. The results indicate that both the elastic modulus and the compressive yield strength increase significantly with increasing strain rate at all temperatures analyzed, and that the deformation process shows a limitation in the localization of plastic deformation within the epoxy material at higher ambient temperature. Furthermore, significant changes were observed regarding the improvement of compressive strength properties near the glass transition temperature under dynamic loading conditions.

An analysis of the resin’s mechanical properties in study [42]—such as the modulus of elasticity, yield strength, and ultimate tensile strength—revealed that the behavior of the epoxy polymer depends significantly on the strain rate. Three-point bending (TPB) tests were conducted under both quasi-static (1–10 mm/min) and dynamic (10–15 m/s) conditions. It was found that fracture toughness increases with displacement rate in the quasi-static regime, a result of significant plastic deformation. Conversely, under dynamic loading conditions, a decrease in this value was observed, attributed to the absence of plastic deformation and the occurrence of brittle fracture. Meanwhile, the tensile strength investigated in study [43] decreased linearly with increasing temperature, whereas it increased logarithmically with increasing loading rate. In summary, when a destructive force acts slowly, the resin’s polymer chains have time to relax and undergo spatial rearrangement. Plastic deformation absorbs a portion of the energy, resulting in a rough, matte fracture surface. Most researchers indicate that fracture toughness is higher in this case because a significant amount of energy is dissipated as heat and plastic deformation. Under dynamic conditions, however, plastic deformation of the polymer network does not occur; the resin behaves as a perfectly elastic material and is susceptible to sudden fracture at lower energy levels. Fracture toughness drops markedly, and the resulting fracture surface is smooth, sometimes displaying visible striations.

The aim of this study is to investigate the effect of velocity and lay-up of tested carbon–epoxy composites on the amount of energy absorbed during the progressive crushing process.

## 2. Materials and Methods

For this study, tubular specimens with an internal diameter of 20 mm and a height of 34 mm were prepared. The wall thickness for each specimen was 2 mm. Prepregs from Impregnatex Compositi were used to create specimens with different architectures: plain weave (WF) and unidirectional 3k (UD). Combining the number of layers of specific prepregs allowed for different proportions of axial fibers to peripheral fibers. The properties of the finished composite were determined experimentally and are shown in Table 2. The properties of the resin are given in Table 3.

**Figure 1:**
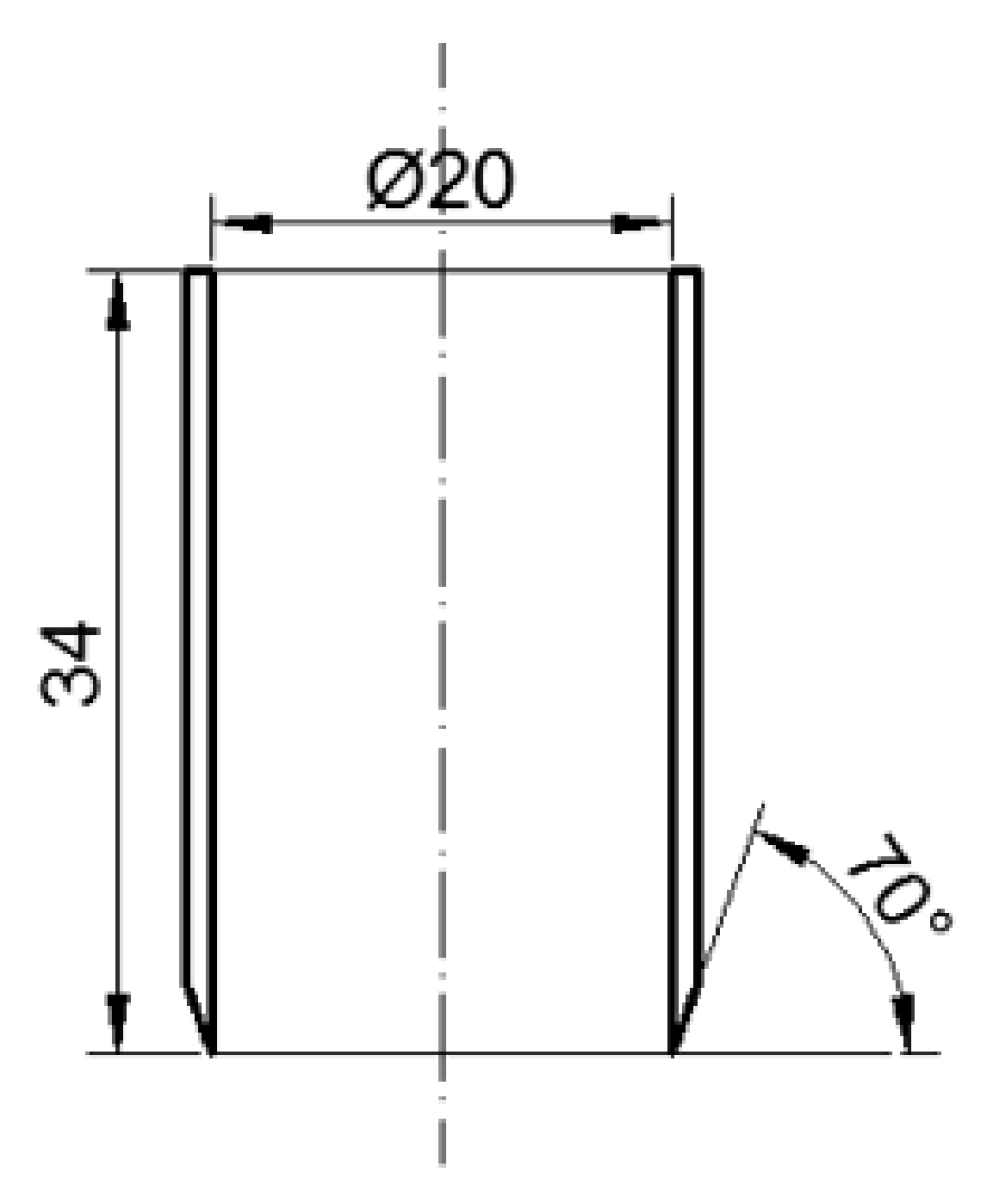
Geometry of tube [38].

**Figure 2:**
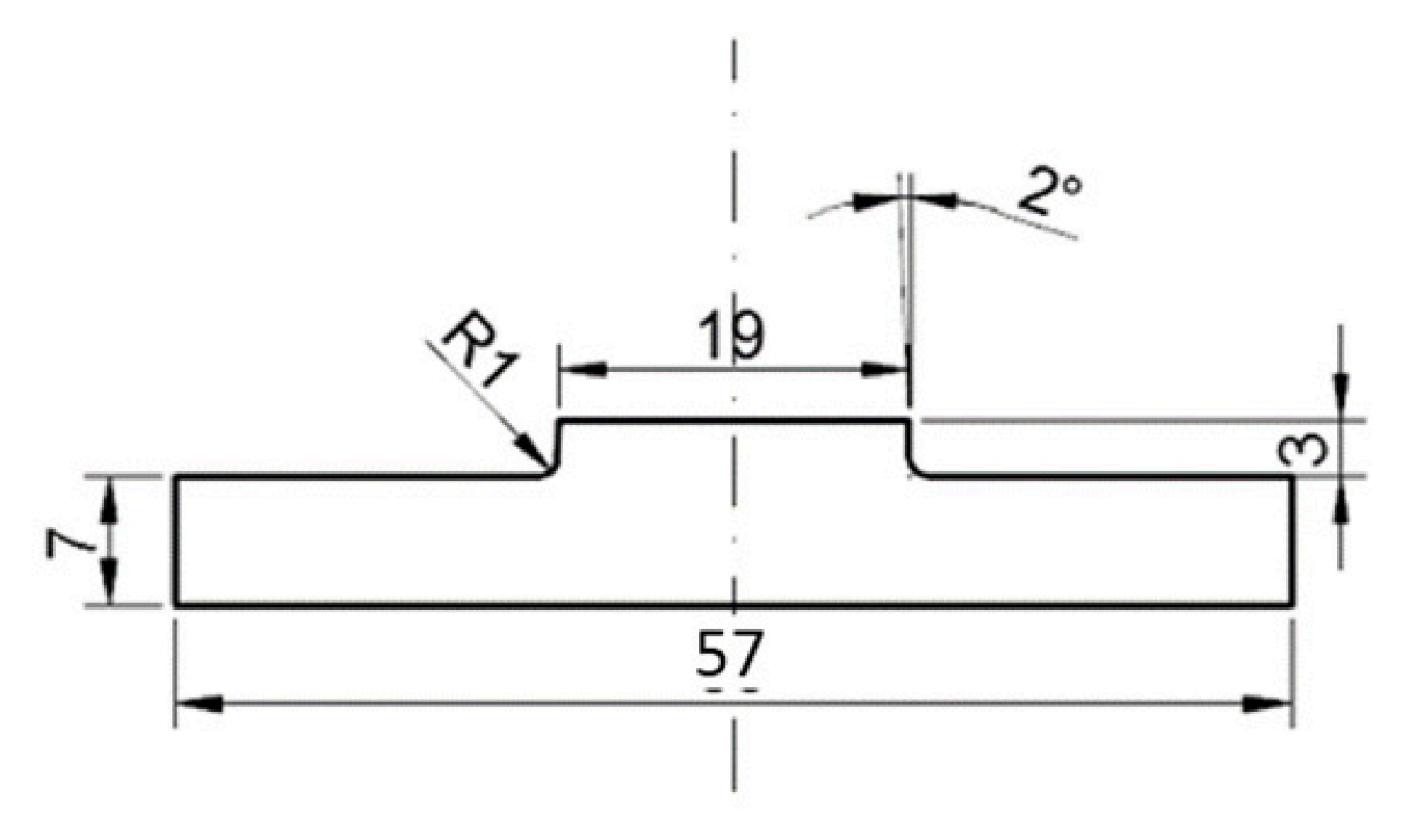
Plug initiator geometry [38].

**Figure 3:**
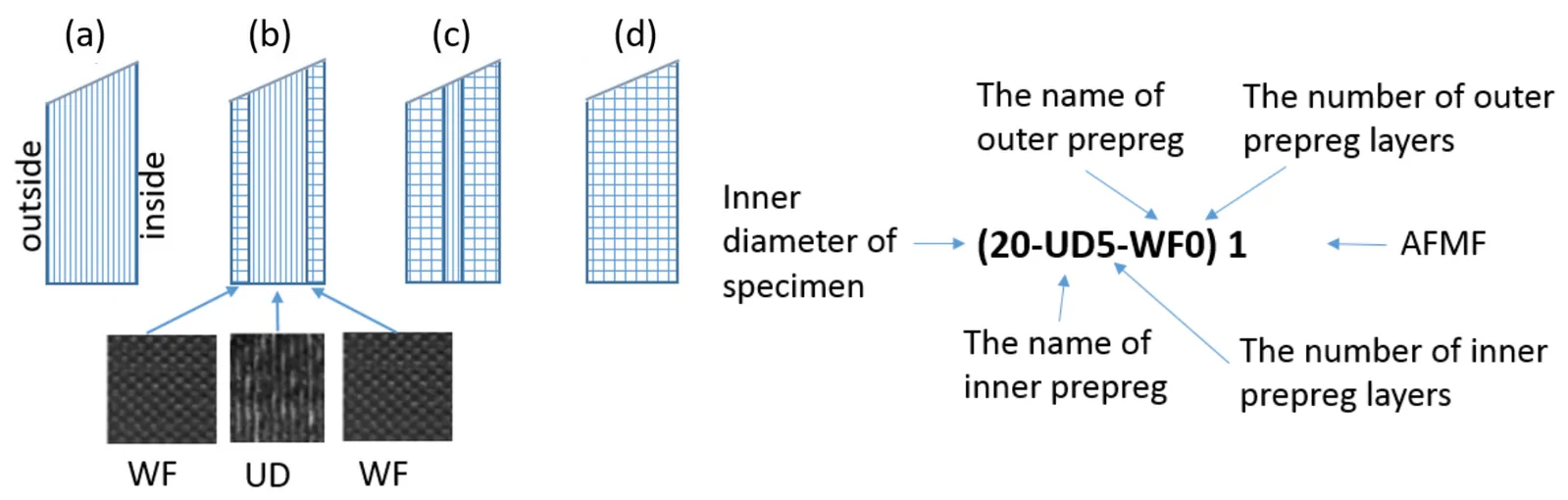
The specimen architecture: (**a**) (20-UD5-WF0) 1; (**b**) (20-UD3-WF2) 0.8; (**c**) (20-UD1-WF4) 0.6; (**d**) (20-UD0-WF5) 0.5.

Specimens were made by manually applying prepreg layers onto a Teflon core and then cured in an autoclave. A representative specimen is shown in Figure 1. An initiator with a working radius of 1 mm was used for the tests, and each specimen had a 70° chamfer as a trigger (Figure 2). The tube specimen configurations were designed to differ in terms of AFMF values (axial fiber mass to total fiber mass) [24]. This effect was achieved by using a different number of UD layers in relation to the number of WF layers, as shown in Figure 3.

### 2.1. Quasi Static Test

**Figure 4:**
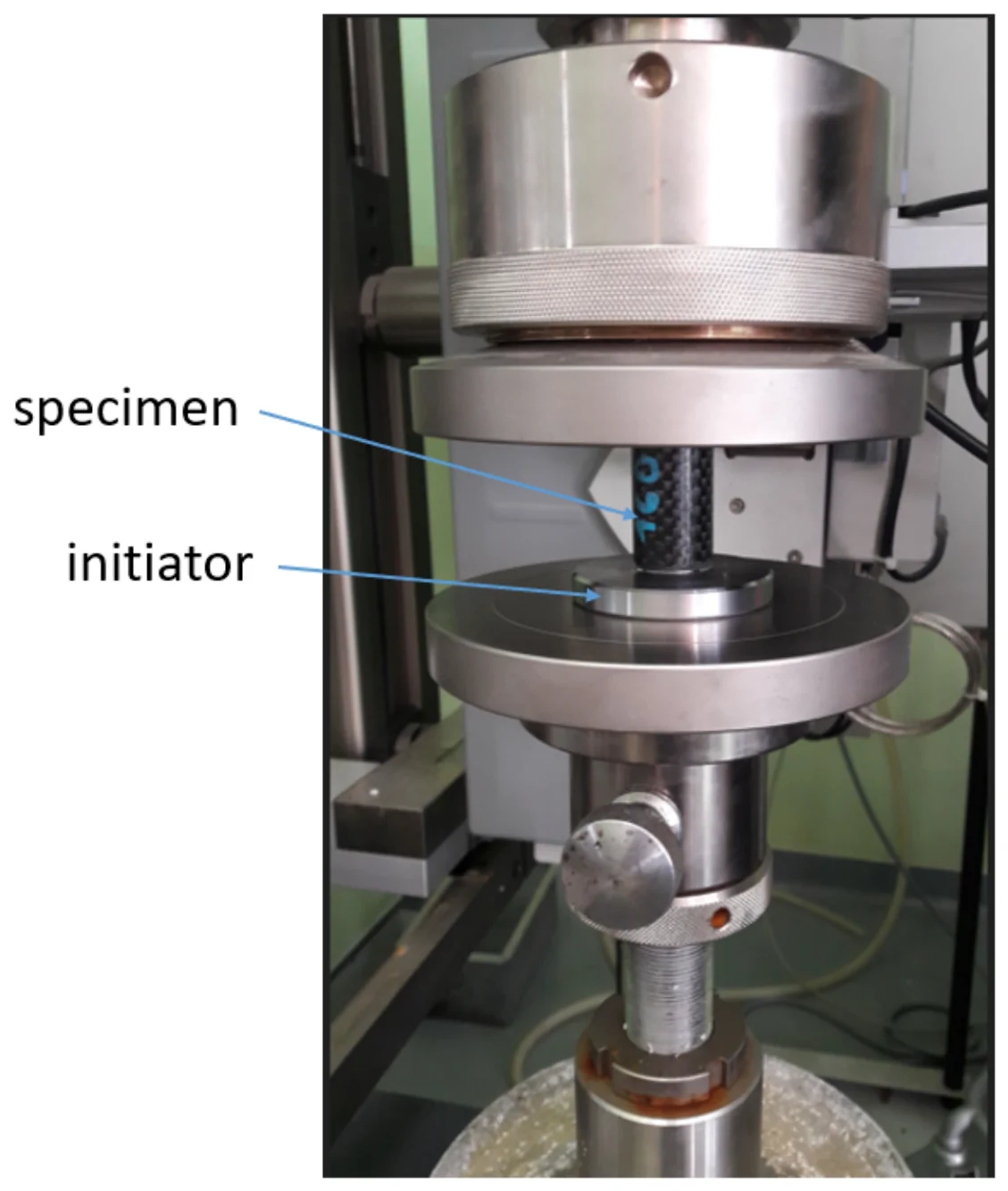
Test stand for performing compression tests under quasi-static conditions, v = 20 mm/min (QS) [38].

A Zwick 30 kN testing machine was used in the tests (Figure 4). The process velocity was constant and equal to v = 0.0003 m/s, and the displacement for each specimen was 25 mm.

**Figure 5:**
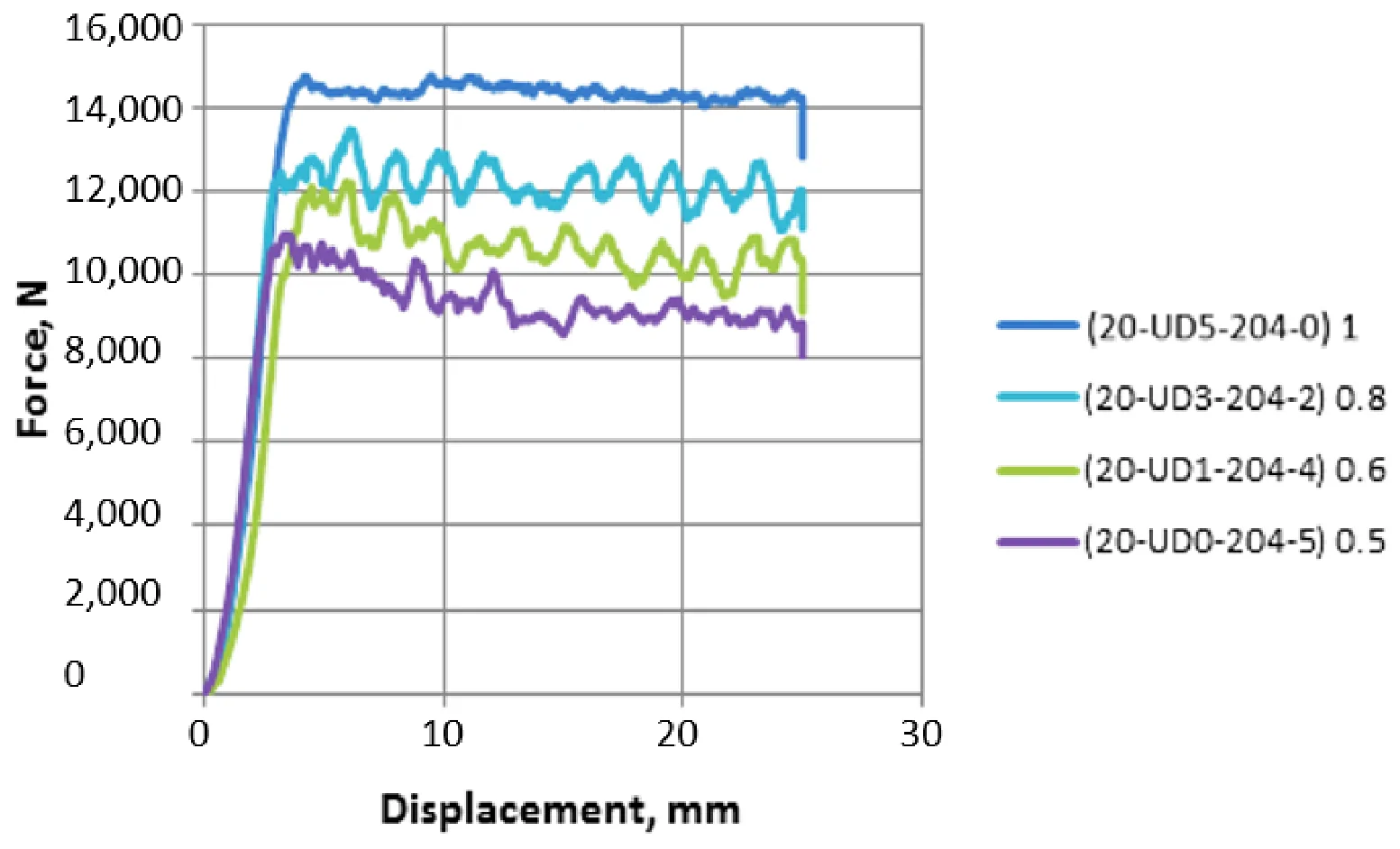
Example compression test results for different AFMFs—quasi-static conditions (QS).

**Figure 6:**
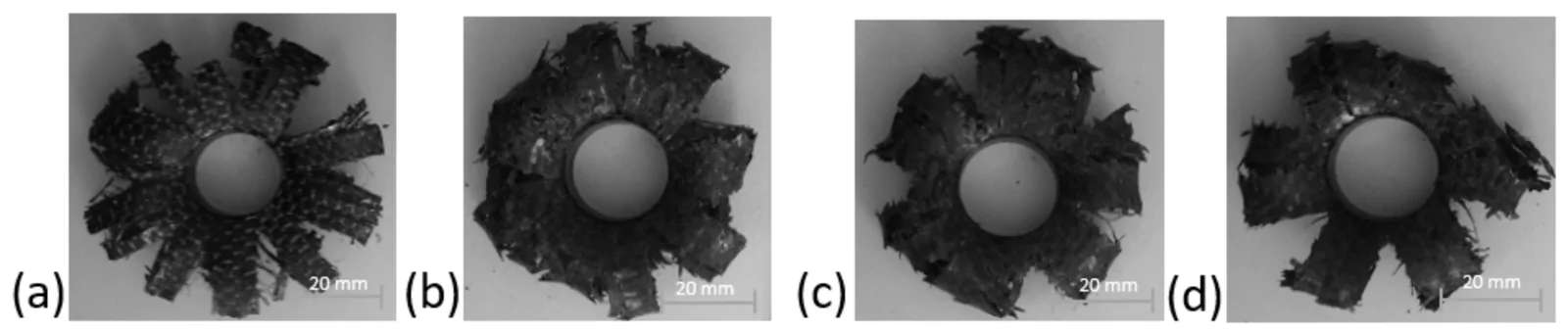
Example view of specimens after test; quasi-static test 20 mm/min (QS); (**a**) AFMF = 1; (**b**) AFMF = 0.8; (**c**) AFMF = 0.6; (**d**) AFMF = 0.5 [38].

During the tests, force and displacement were recorded under quasi-static conditions. Based on the obtained results, graphs were created for all specimens (Figure 5). The appearance of the specimens after testing is shown in the photos (Figure 6). 

### 2.2. Dynamic Test

**Figure 7:**
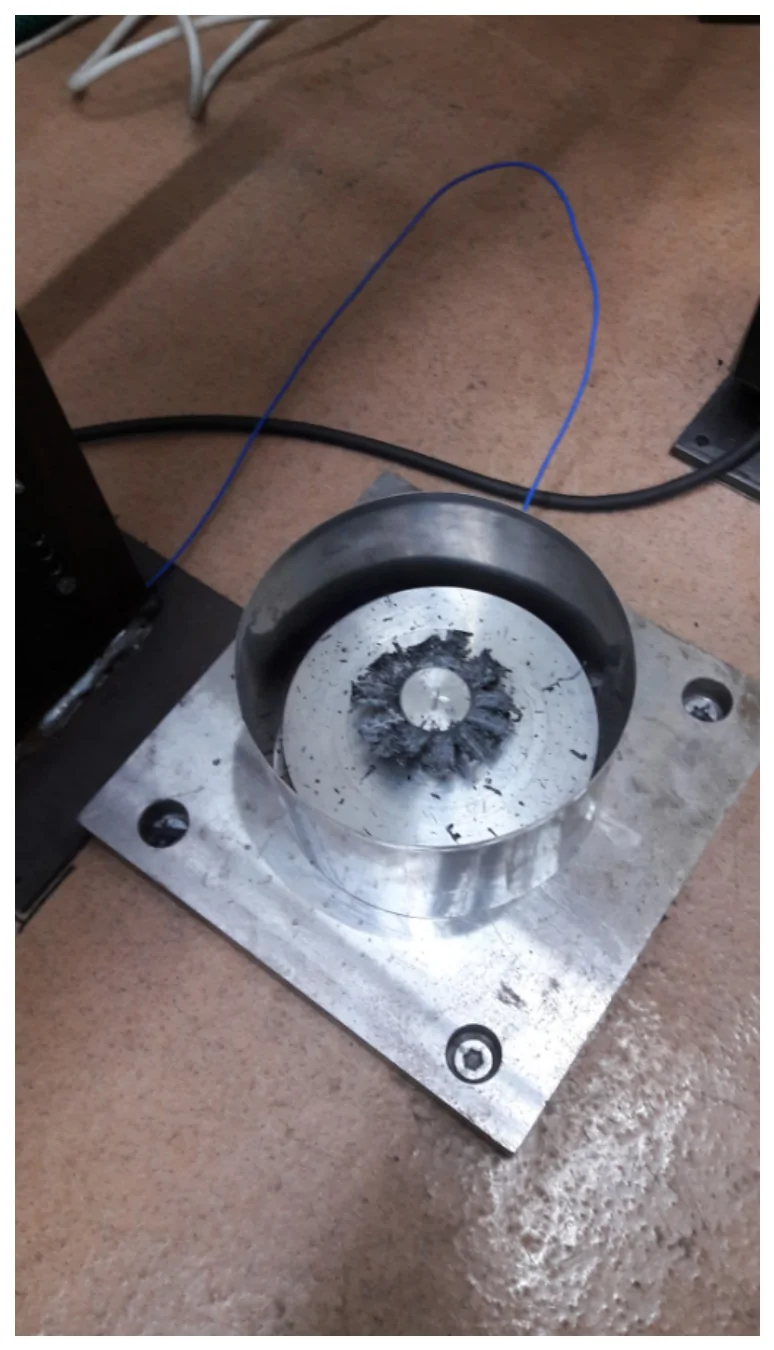
View of the specimen after the test (D).

Dynamic tests (D) were conducted using a gravity hammer. The test specimens differed in their configuration (AFMF), which resulted in different energy absorption rates. This necessitated the adjustment of the hammer’s energy by adjusting the drop height. The collected force and displacement data allowed for the generation of force-versus-displacement graphs. The view of the specimen after the test is shown in Figure 7.

**Figure 8:**
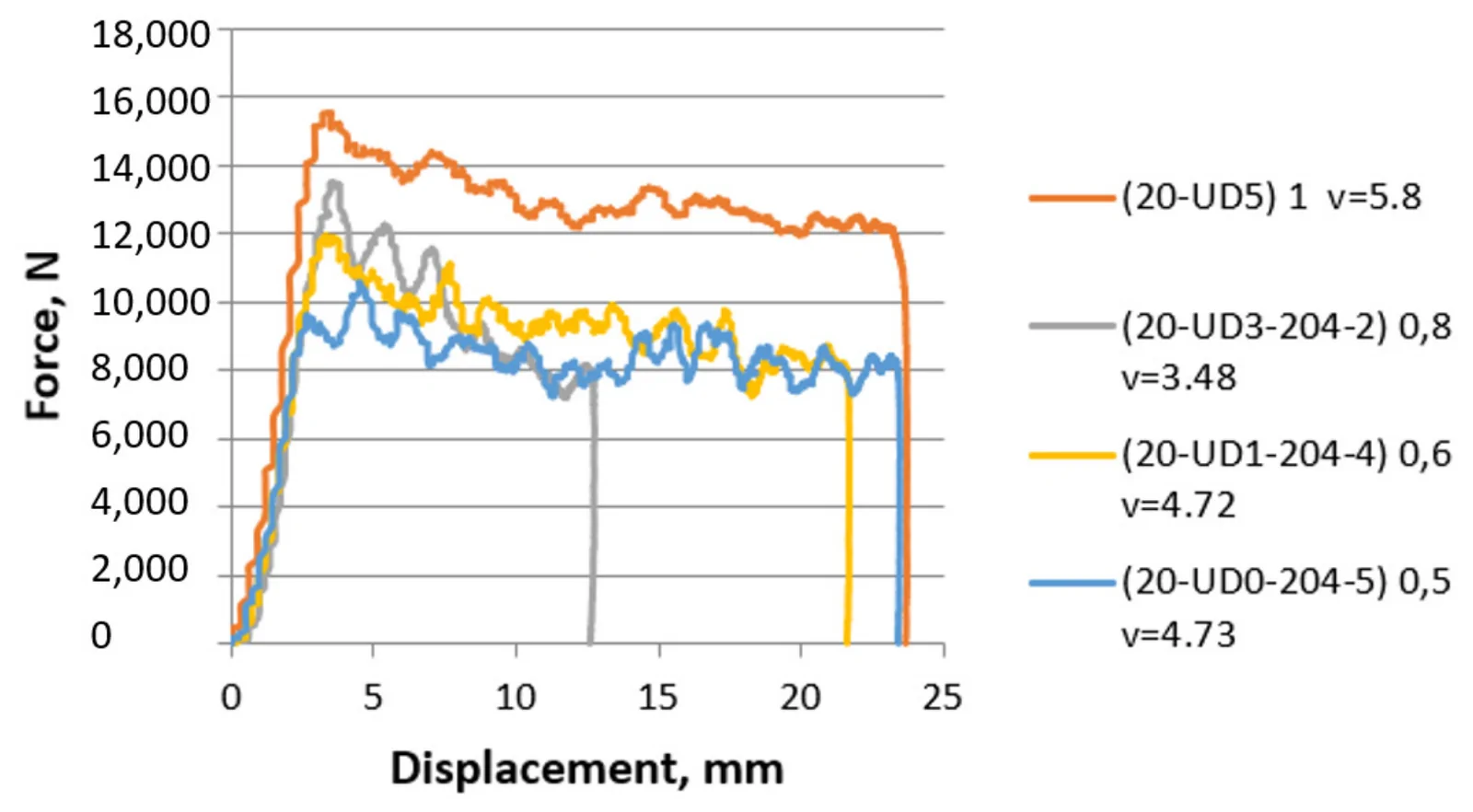
Force displacement curve for dynamic conditions (D); results for example specimens.

**Figure 9:**
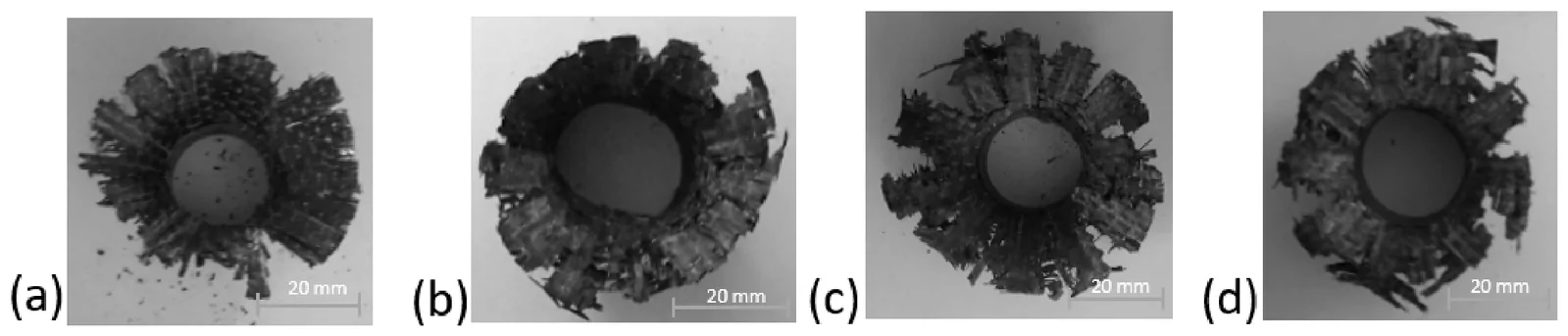
Representative view of crushed specimens; dynamic conditions (D); (**a**) AFMF = 1; (**b**) AFMF = 0.8; (**c**) AFMF = 0.6; (**d**) AFMF = 0.5 [38].

Experimental compression tests on composite specimens yielded force-displacement curves (Figure 8). Figure 9 shows sample photographs of the specimens following the experimental tests.

As the proportion of axial fibers in the samples decreases, more intense fiber breakage is observed, which may indicate the dominance of the fragmentation mechanism. It is worth noting that the shape of the plots shown in Figure 8 (dynamic conditions) differs significantly from those in Figure 5 (quasi-static conditions). The plots in Figure 8 are more irregular, and the displacement value depends on the impact energy of the drop hammer. The higher the AFMF value, the greater the contribution of the delamination mechanism (Figure 9). At low AFMF values, fragmentation predominates.

Transverse fracture of the axial fibers appears to be the primary mechanism responsible for energy absorption during compression. This phenomenon is most pronounced at high AFMF values. It is facilitated by the action of an initiator with a small working-edge radius, the strength of the resin binding the axial fibers, and the absence of circumferential fibers. Because the transverse fracture of axial fibers proceeds with high intensity, it constitutes the dominant mechanism for energy absorption by the composite structure.

### 2.3. Split Hopkinson Pressure Bar Test

**Figure 10:**
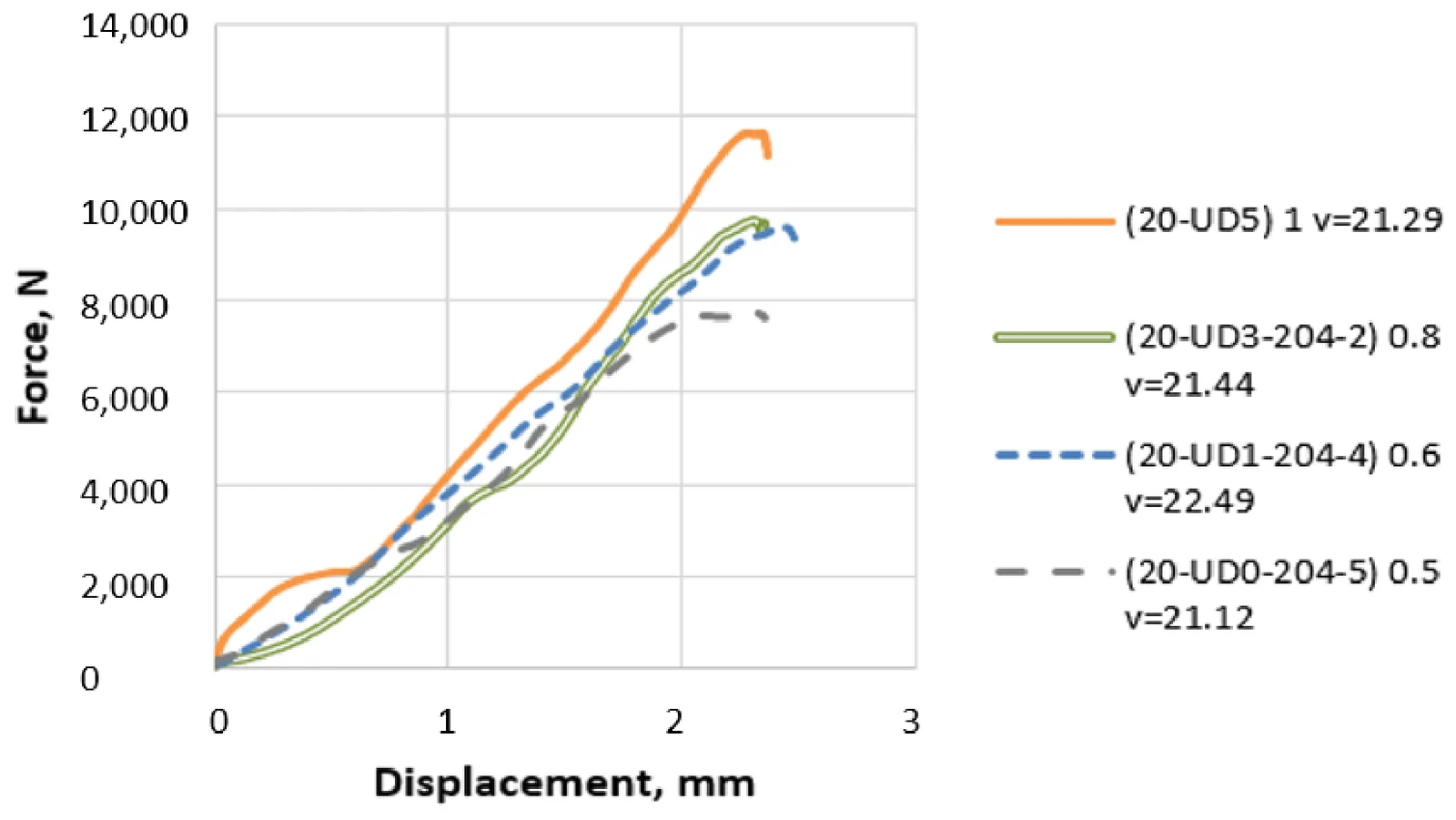
Force–displacement curve for SHPB tests; results for example specimens.

**Figure 11:**
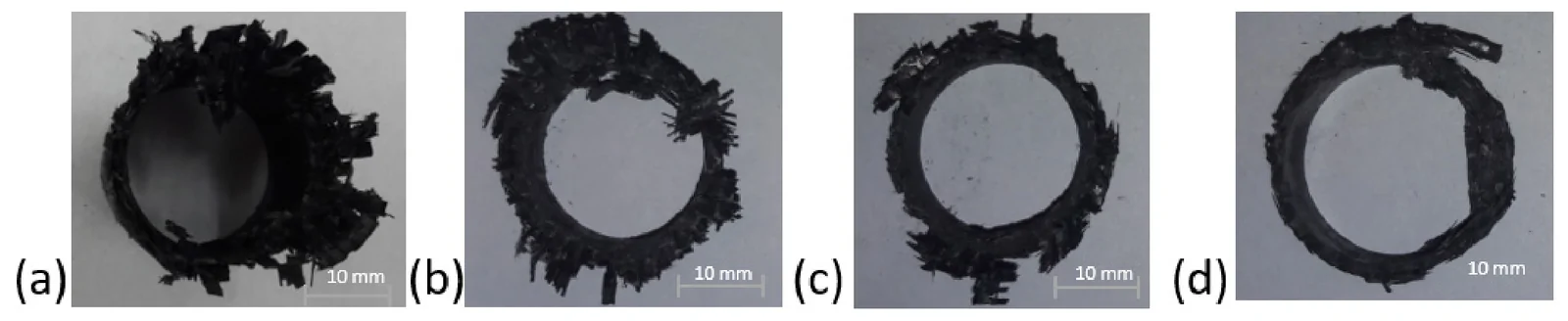
Representative view of crushed specimens; SHPB test; (**a**) AFMF = 1; (**b**) AFMF = 0.8; (**c**) AFMF = 0.6; (**d**) AFMF = 0.5.

The specimens for the SHPB tests were shortened to 18 mm due to the type of test. Details of the measurement technique are presented in [44,45]. The obtained measurement values were used to generate force versus displacement graphs (Figure 10). Three specimens were tested for each AFMF value. The representative views of crushed specimens are shown in Figure 11.

The use of SHPB testing did not allow for the determination of the full-range force–displacement relationship. Observations and conclusions drawn from the conducted tests are limited compared to results obtained at lower speed. However, it can be stated that the amount of absorbed energy increases with rising AFMF. This trend was observed across all tested samples and test conditions in this study. Delamination was observed to a lesser extent than in tests conducted at lower speed; however, it is more pronounced at high AFMF values and diminishes as AFMF decreases, giving way to a fragmentation mechanism.

## 3. Discussion

Specific energy absorption was calculated as

(1) $$SEA=E_{tot}/\rho Al$$

where E_tot_—energy absorbed by the structure;

ρ—mean density of the tube;

A—cross-sectional area of the tube;

l—crushed stroke.

**Figure 12:**
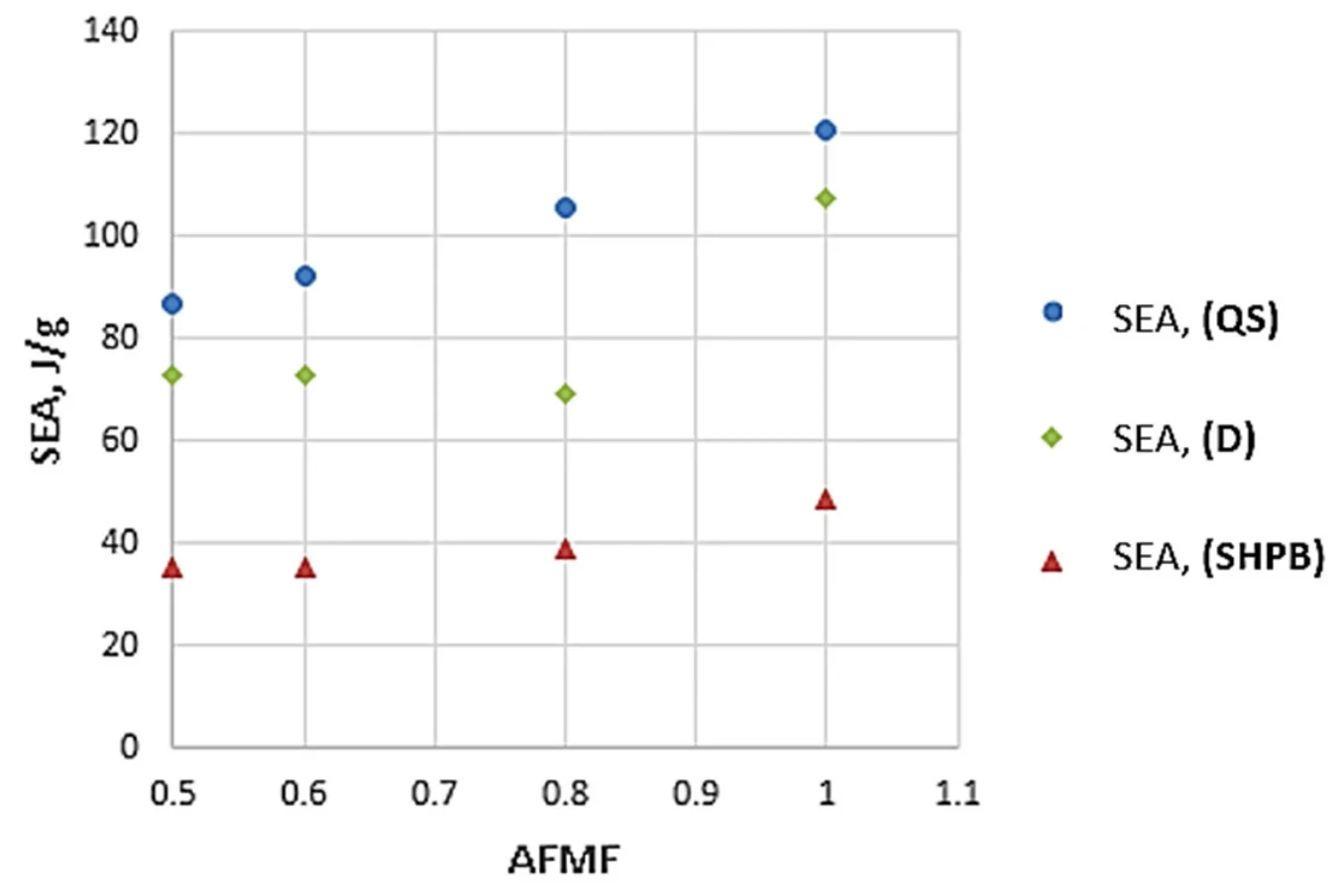
SEA as a function of the AFMF. Results for quasi-static test (QS), dynamic test (D) and SHPB test.

In order to compare how velocity affects the amount of absorbed energy, the results of the experiment performed under quasi-static, dynamic, and SPHB conditions were compared (Figure 12).

The experimental studies presented in this paper indicate that an increase in test speed results in a reduction in the energy absorbed during the compression of the composite (Table 4).

**Figure 13:**
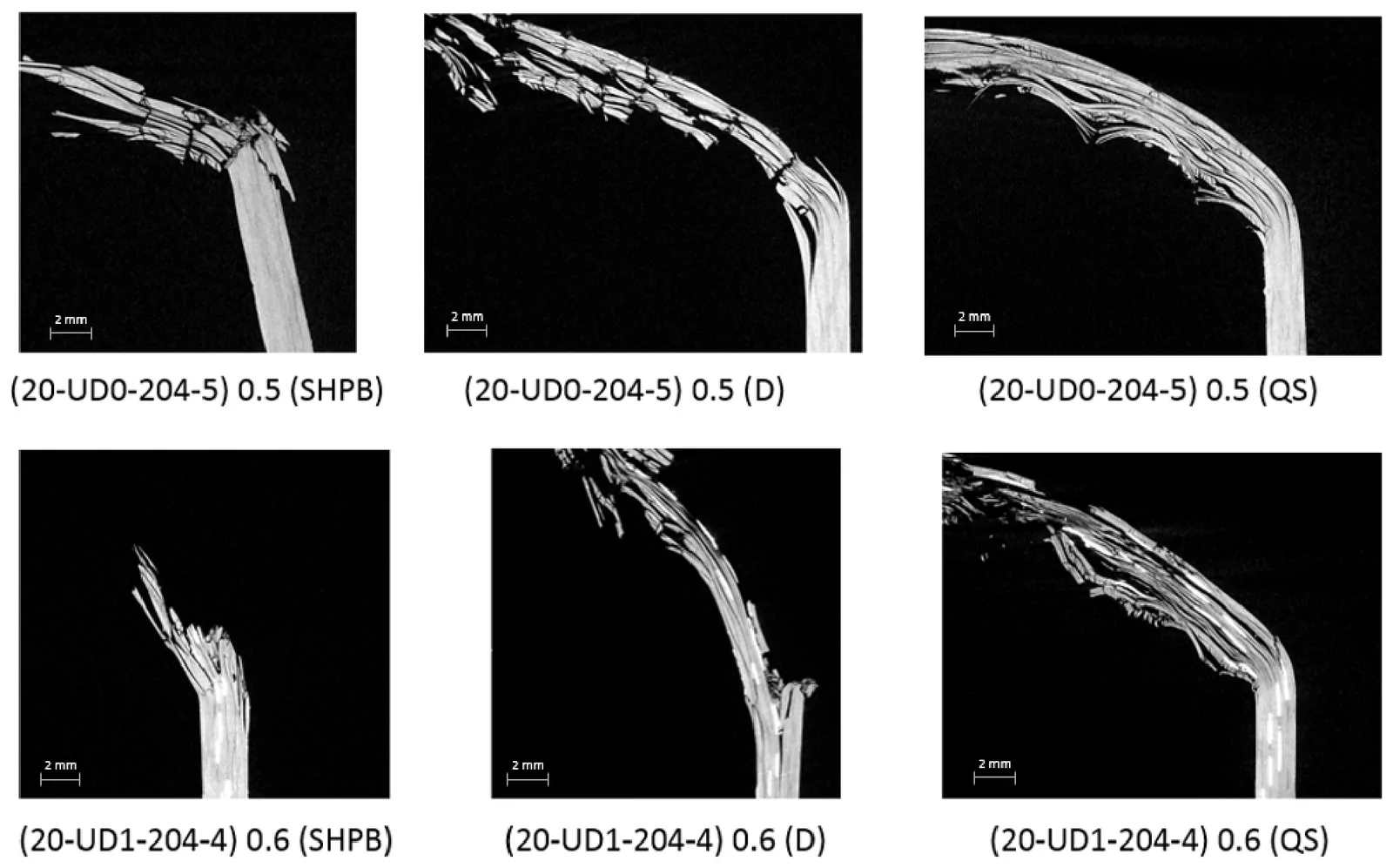

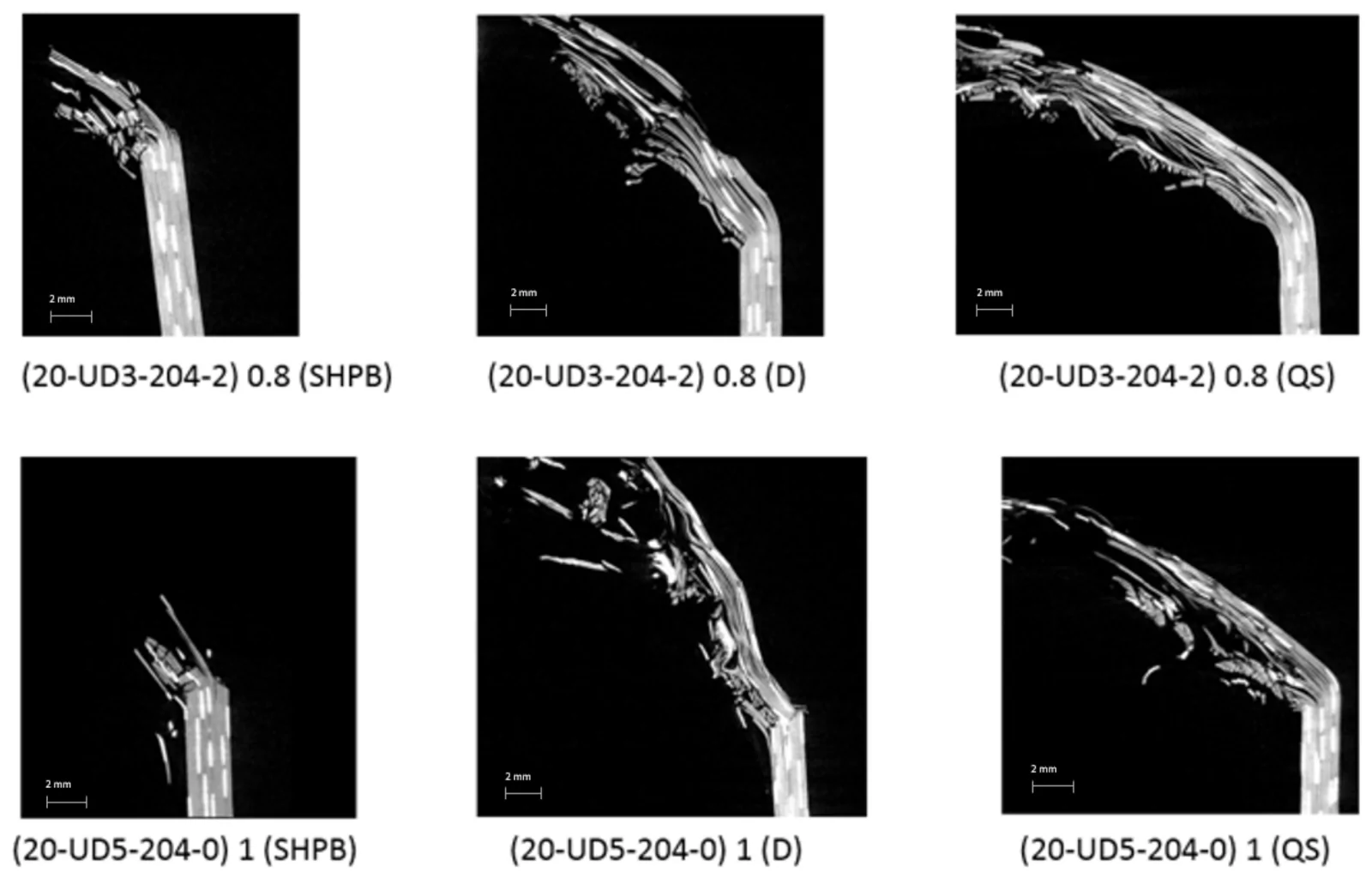
View of specimens with different architecture (AFMF = 0.5; 0.6; 0.8; 1) after crushing in quasi-static (QS) and dynamic (D) conditions and with the use of Split Hopkinson Pressure Bar (SHPB).

Computed tomography (CT) enables insight into the structure of specimens after testing and can also help identify energy absorption mechanisms occurring during the crushing process, depending on the crushing velocity used. Measurements were made on the Nanotom S, a General Electric device. Representative photos are shown in Figure 13.

**Figure 14:**
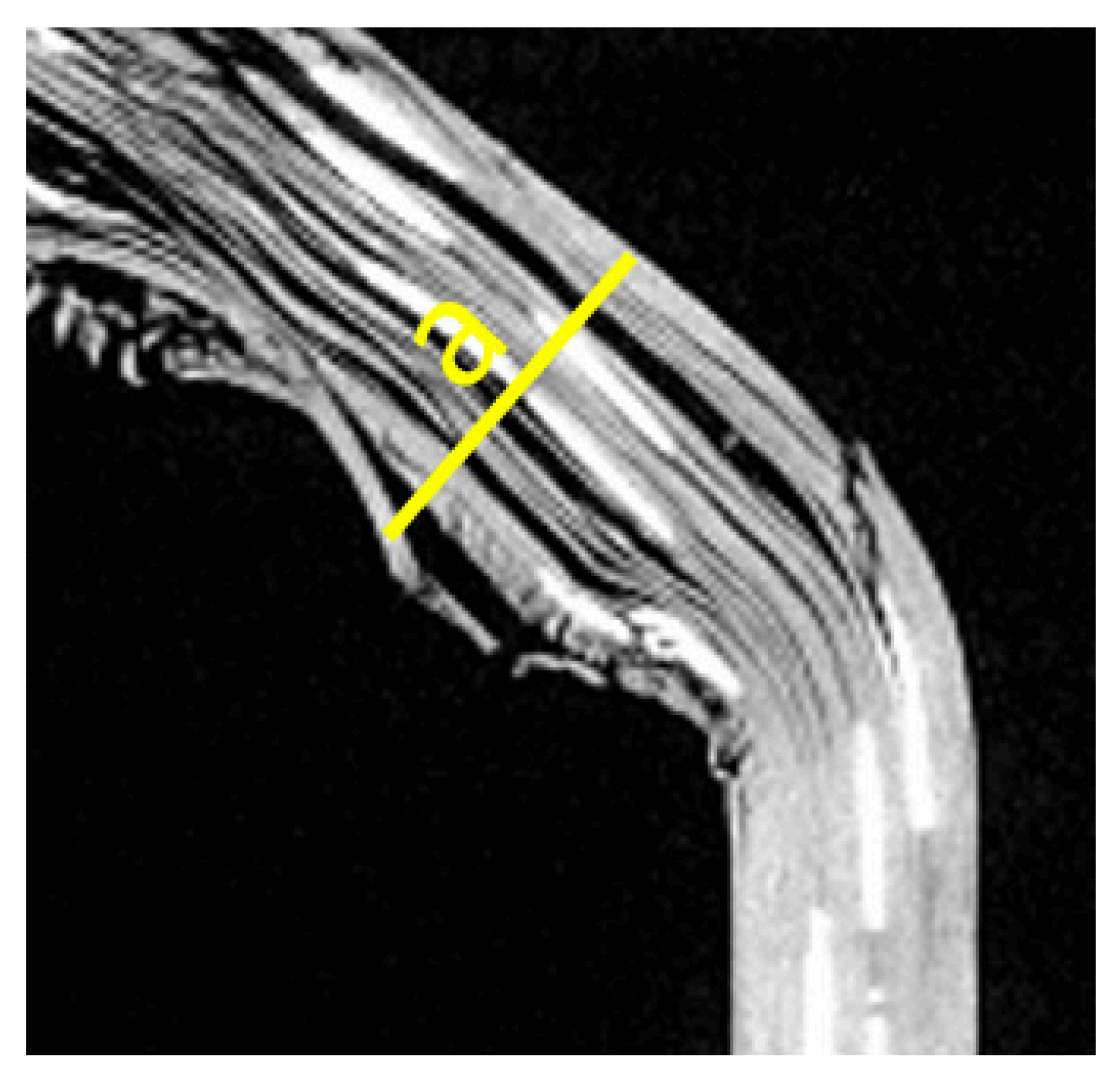
(20-UD5-204-0) (QS) AFMF = 1, method of measurement (plot profile).

**Figure 15:**
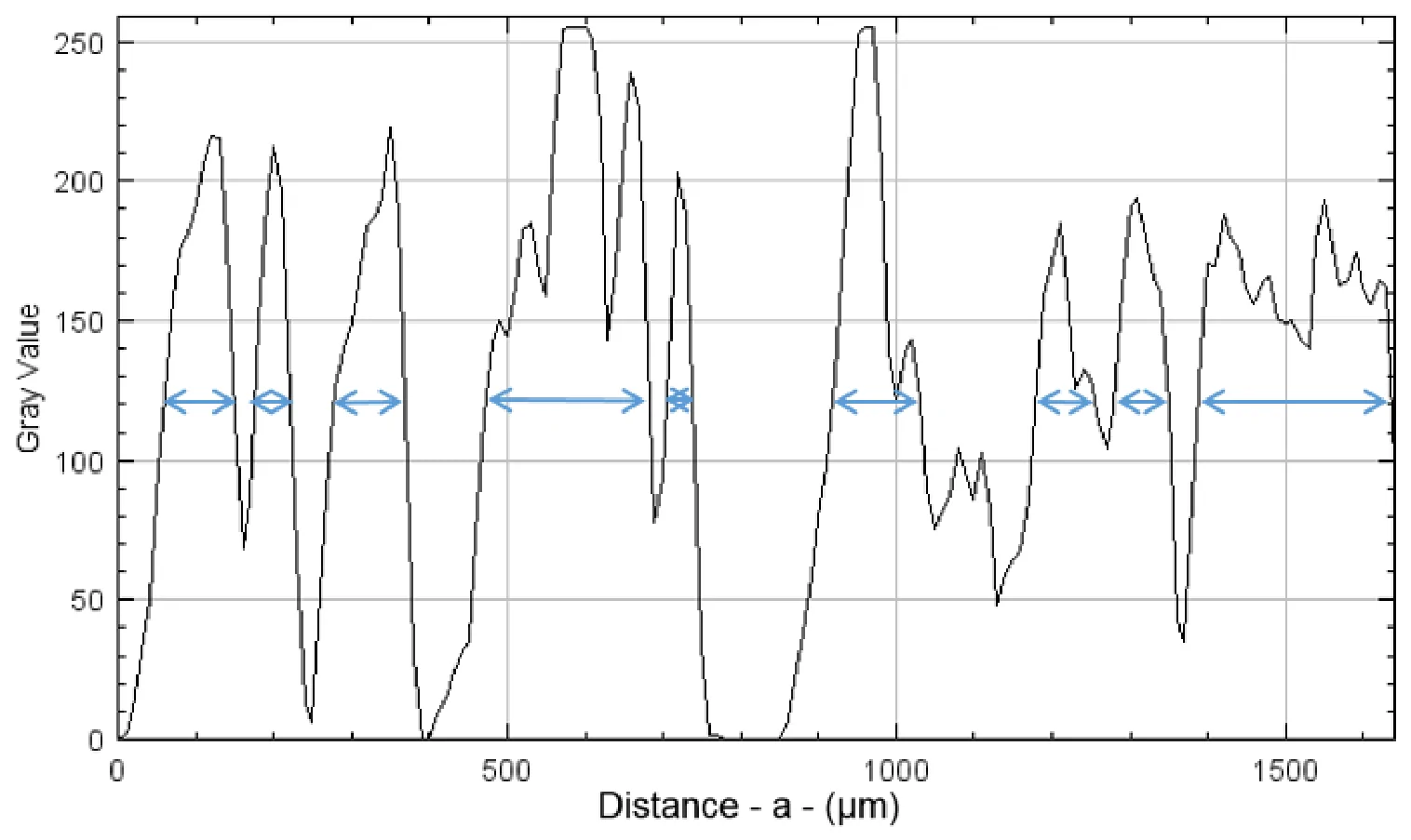
(20-UD5-204-0) (QS) AFMF = 1, plot profile.

In order to determine the thickness of the layers in the specimen after the crushing process, the measurement place (Figure 14) was determined, and then, using the image analysis function in Fiji Is Just ImageJ software 1.54J, plot profiles were made for the analyzed representative specimens. An exemplary plot profile for the specimen (20-UD5-204-0) (QS) is shown in Figure 15.

**Figure 16:**
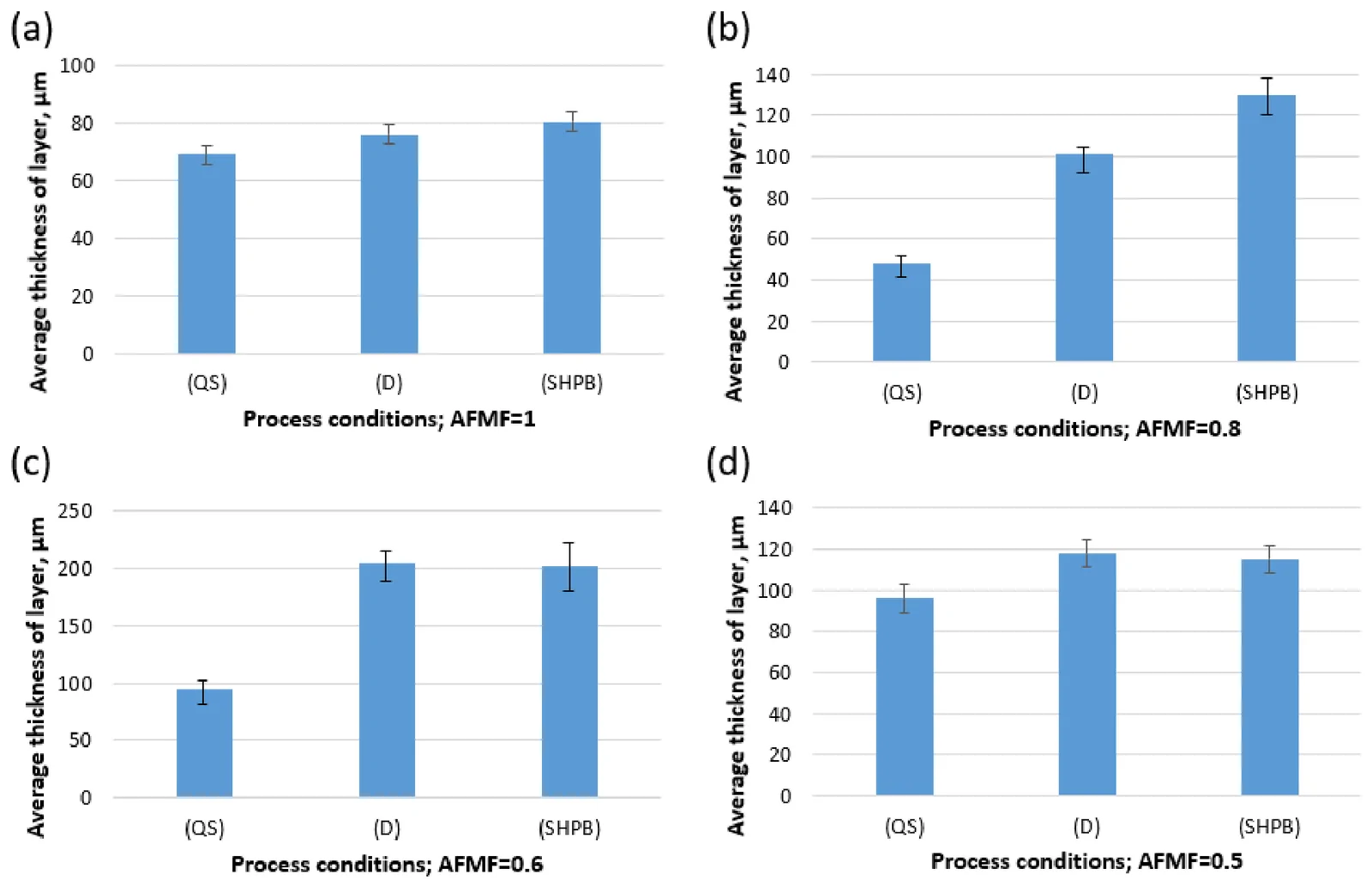
(**a**) Average layer thickness after crushing for AFMF = 1 and various process velocity; (**b**) average layer thickness after crushing for AFMF = 0.8 and various process velocity; (**c**) average layer thickness after crushing for AFMF = 0.6 and various process velocity; (**d**) average layer thickness after crushing for AFMF = 0.5 and various process velocity.

**Figure 17:**
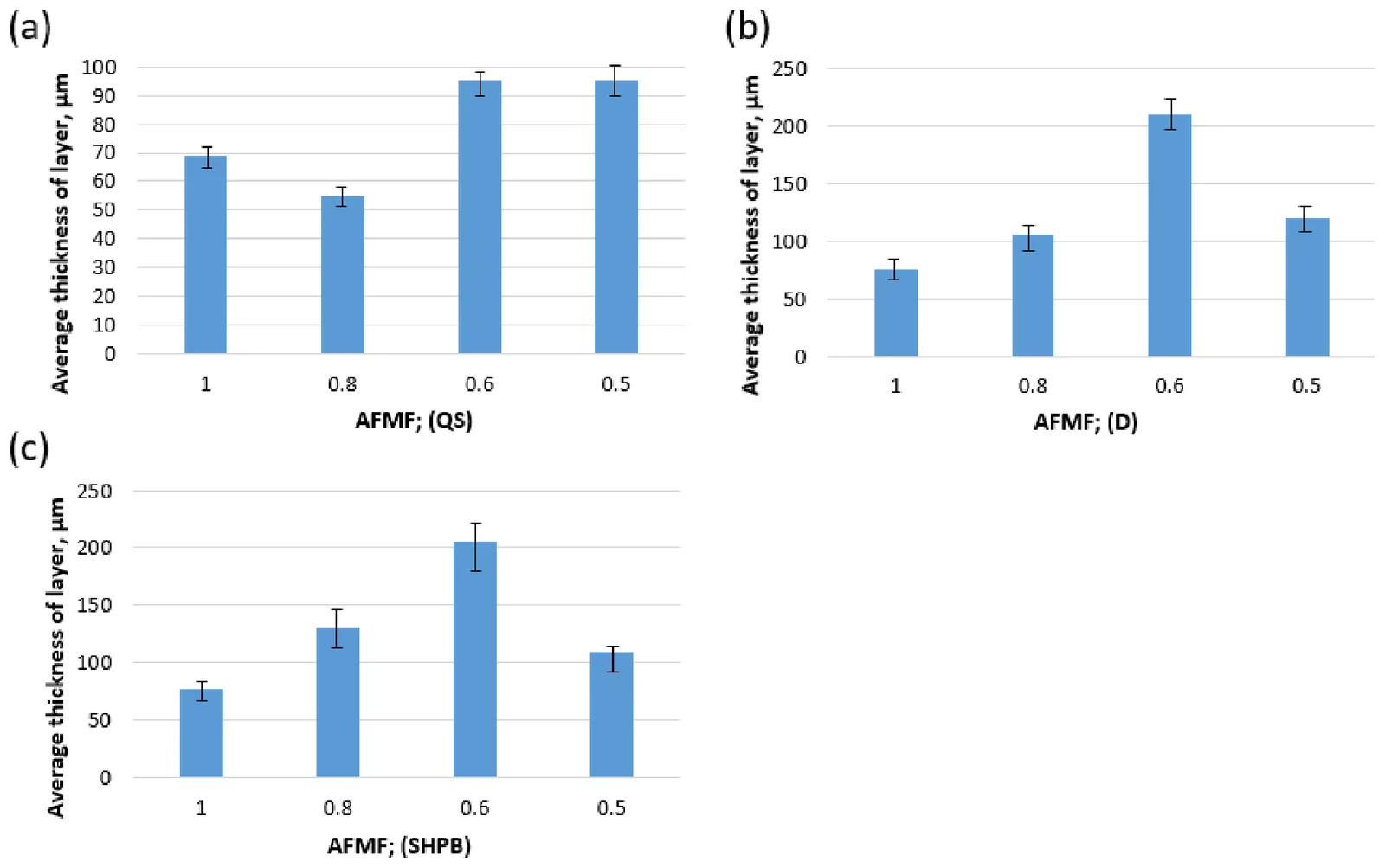
(**a**) Average layer thickness after crushing under quasi-static conditions (QS) for specimens with different AFMFs; (**b**) average layer thickness after crushing under dynamic conditions (D) for specimens with different AFMFs; (**c**) average layer thickness after crushing using SHPB for specimens with different AFMFs.

All CT scans were formatted in the same way (brightness, contrast); then, using plot profiles for gray value equal to 120 (mean gray value obtained), the average layer thickness was determined for each specimen (depending on the process velocity and architecture). The results are presented in Figure 16 and Figure 17.

The analysis of the obtained results leads to the conclusion that the increase in the process velocity causes the increase in the average layer thickness after the crushing process. This indicates a change in the energy absorption mechanism in this process depending on the velocity. For the quasi-static process, intense separation of the composite into thin layers can be observed. This mechanism, in addition to the fiber bending and breaking mechanism on the initiator radius, which was described in [24], seems to be dominant in quasi-static conditions, leading to the absorption of the highest energy in this test (Figure 16 and Figure 17a). As the test velocity increases, the process of intensive separation into layers decreases (the average layer thickness increases) and the amount of absorbed energy decreases. Figure 17 shows the dependence of the average layer thickness depending on the specimen architecture.

The smallest layer thickness occurs for specimens containing only axial fibers (AFMF = 1). As the share of axial fibers decreases (decreasing AFMF), the average layer thickness increases (the share of the delamination mechanism decreases), and thus the amount of absorbed energy decreases.

## 4. Conclusions

Based on the conducted research, it can be concluded that the axial fiber content is a crucial factor influencing the energy absorption, peak force, and steady-state force during the compression of tubular CFRP composites. This relationship was observed across the wide range of test speeds investigated (20 mm/min to 21 m/s). The higher the axial fiber content, the greater the specific energy absorption (SEA).

Increasing the test speed from 20 mm/min to 21 m/s results in a 59.3% decrease in absorbed energy when the ratio of circumferential to axial fibers is equal (AFMF = 0.5). When the axial fiber content was increased from 50% to 100%, an increase in absorbed energy of 39% was observed for low test speed (quasi-static conditions) and 46.85% for dynamic conditions. At high test speed (using the Hopkinson bar), increasing the axial fiber content resulted in a rise in SEA of approximately 37.58%.

The relationship between the amount of energy absorbed and the layer thickness after the crushing process, depending on the process velocity and specimen’s architecture, was shown. Increasing the test velocity increases the average layer thickness after the process. Increasing the proportion of axial fibers (AFMF) reduces the average layer thickness after the process.

## Figures and Tables

**Table 1:** Exemplary data showing reduction or increase in SEA depending on the test velocity.

Ref.	Specimen Type	Velocity, m/s	SEA Change
[5]	CF/epoxy, tube ○ [±75]_3_	1 × 10^−4^ ÷ 12	+35%
[14]	CF/epoxy, tube ○	0.001 ÷ 12	−20%
[17]	CF/epoxy, tube ○ [0, ±90]	8.3 × 10^−5^÷ 0.005	+12%
[18]	CF/epoxy, tube ○ [0, ±75]_2_ and [0, ±45]_2_	2 × 10^−6^ ÷ 4 × 10^−5^	0%
[18]	GF/polyester, bar ● (φ5 mm)	2 × 10^−6^ ÷ 4 × 10^−5^	+24%
[19]	GF/CF/polyester, tube □	8.3 × 10^−5^ ÷ 0.008	+9%
[20,21]	GF/epoxy, tube □, cone	2 × 10^−4^ ÷ 8	−15–+20%
[22]	CF/epoxy [(0, ±90)_2_/0/(90, ±0)_2_], DLR	0.001 ÷2	−22%
[23]	CF/epoxy, plate	2 × 10^−5^ ÷5.2	−19%
[24]	CF/epoxy, tube ○ [0, ±90]	0.0003÷6.7	−10%÷−20%

**Table 2:** Properties of carbon fiber–epoxy composite [38].

	204 3k (WF) Plain Woven	200 3k (UD) Unidirectional
Ply thickness, mm	0.23	0.23
Dry areal density, g/m^2^	204	200
Axial modulus E_1t,_ MPa	50,439.5	104,634.0
Transverse modulus E_2t,_ MPa	49,888.4	9914.0
Strength σ_1t,_ MPa	654.7	1723.0
Strength σ_2t,_ MPa	679.1	78.6
Failure strain ε_1t,_ %	1.23	1.48
Failure strain ε_2t,_ %	1.27	0.81
Axial modulus E_1c,_ MPa	51,526.5	96,983
Transverse modulus E_2c,_ MPa	48,528.0	9489.1
Strength σ_1c,_ MPa	365.3	534.8
Strength σ_2c,_ MPa	317.6	96.4
Failure strain ε_1c,_ %	0.0086	0.0055
Failure strain ε_2c,_ %	0.0081	0.0281
ν_12c_	0.0808	0.2643
ν_21c_	0.0886	0.0178

**Table 3:** Resin matrix properties (from the manufacturer).

	Units	
Density	gr/cm^3^	1.18
Gel Time at 125 °C	min	13.5 ± 3
Tg (DSC)	°C	120÷130
Flexural Strength	MPa	130 ÷ 150
Modulus	MPa	3500 ÷ 4000
Tensile Strength	MPa	45 ÷ 60
Elongation (ISO 527)	%	2.0 ÷ 3.0
Water absorption 60 min 100 °C (212 °F) % 0.2 ÷ 0.5	%	0.2÷0.5

**Table 4:** Summarized results for quasi-static test (QS), dynamic test (D) and SHPB test.

	AFMF	1	0.8	0.6	0.5
QS	Number of specimens	3	3	3	3
	Average velocity, m/s	0.0003	0.0003	0.0003	0.0003
	SEA _QS,_ J/g	121 ± 4	105 ± 4	91 ± 9	86 ± 6
	$\dot{\varepsilon }$, s^−1^	0.0088	0.0088	0.0088	0.0088
D	Number of specimens	3	3	3	3
	Average velocity, m/s	5.03	3.48	4.31	4.32
	SEA _D_, J/g	107.2 ± 8.1	69.4 ± 6.9	73.1 ± 3.4	73.0 ± 5.8
	$\dot{\varepsilon }$, s^−1^	147.9	102.3	126.8	127.1
SHPB	Number of specimens	3	3	3	3
	Average velocity, m/s	21.3	21.41	22.39	21.28
	SEA _SHPB_, J/g	49 ± 5	38.7 ± 0.1	35 ± 4	35 ± 4
	$\dot{\varepsilon }$, s^−1^	1183.3	1189.4	1243.9	1182.2

## Data Availability

The original contributions presented in this study are included in the article. Further inquiries can be directed to the corresponding author.
